# Lab-on-a-tip platform with cotton matrices for colorimetric detection of carmine in lipsticks

**DOI:** 10.1039/d6ra03072f

**Published:** 2026-07-03

**Authors:** Balachandar Sundarrajan, Oyessi Dutta, Sohini Datta, Anusha Prabhu, Lokitha Paduvetnaya, Harishkumar Madhyastha, Naresh Kumar Mani

**Affiliations:** a Manipal Institute of Technology, Manipal Academy of Higher Education Manipal India; b Bioprocess Engineering Laboratory, Department of Chemical Engineering, Birla Institute of Technology and Science Pilani Goa 403726 India; c Department of Cardiovascular Physiology, Faculty of Medicine, University of Miyazaki Miyazaki 889-2192 Japan naresh.mani@manipal.edu maninaresh@gmail.com

## Abstract

Carmine is a natural red dye derived from cochineal insects and is widely used in cosmetics and food products. Since carmine is derived from insects, individuals who follow vegan or vegetarian lifestyles and those who have religious beliefs may wish to ascertain its presence in the products they use. Additionally, carmine causes angioedema, urticaria, contact dermatitis, systemic anaphylaxis and even asthma in hypersensitive individuals. Conventional methods for detecting the presence of carmine require expensive equipment and skilled personnel; thus, robust, low-cost and rapid detection is needed. In this work, we fabricated a simple lab-on-a-tip device with reagents imbibed in two cotton matrices for the colorimetric detection of carmine. In this assay, carmine reacts with hydrochloric acid to produce carminic acid, which further reacts with ferric chloride to form a brown chromogenic complex whose intensity is proportional to the carmine concentration. Additionally, a comparative evaluation was performed between cotton and filter paper, in which cotton exhibited enhanced color intensity and sensitivity because of its three-dimensional intertwined fibrous network. The simple lab-on-a-tip device provides a reproducible and distinguishable color across the selected concentration range, with an experimentally determined limit of detection (LOD) of 0.5 mg mL^−1^. FTIR spectral analysis confirmed the presence of characteristic functional moieties of carmine in commercial lipstick samples, as validated against standard carmine. The device was successfully applied to commercial samples where carmine was detected in some lipstick samples, while no detectable color change was observed in one lipstick sample. Overall, this work presents a simple, portable and cost-effective (USD $ 0.0055) device for onsite and rapid detection of carmine, with a green metric score of 0.74 determined using AGREE software, proving it to be a green method. Compared with conventional techniques, this device has minimal resource requirements and potential for point-of-care and field applications, especially in low-resource settings.

## Abstract 2

Cotton-based lab-on-a-tip device enables colorimetric detection of carmine through formation of a brown ferric–carminic acid complex with a LOD of 0.5 mg mL^−1^. The assay's potential using commercial lipstick underscores a portable low-cost platform.

## Graphical abstract

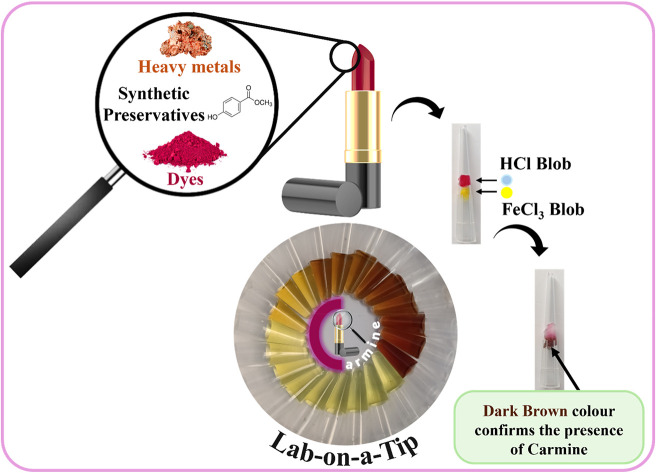

## 1 Introduction

Cosmetics are defined as products that are applied to the human body to improve appearance. Although not essential for physical health, the use of cosmetics has been linked to improving psychological well-being and confidence. Cosmetics have been around for millennia, mostly derived from natural sources such as animals, plants and minerals,^1,2^ and their use has consistently played a crucial role in societal and cultural practices. The use of kohl in ancient Egypt, rice powder in ancient China, turmeric in ancient India, and olive oil in the Mediterranean are examples of how cosmetics have been part of the culture for eons. The 19th and early 20th centuries showed a revolutionary era in the history of cosmetics, during which cosmetics production transformed from homemade concoctions to mass-produced industrial manufacturing, bringing the age of modern beauty.^3^ On the basis of their mode of use, modern cosmetics can be classified as leave-on products that remain on the skin for extended periods, such as lipstick, creams, and body lotions, and rinse-off products that are applied temporarily and then washed away, such as shampoos, gels, and soaps.^4^

With rapid industrialization, the global cosmetics industry has experienced significant growth in recent years. According to Statista, the beauty and cosmetics industry is forecasted to reach nearly 140 billion USD by the year 2030, with the skincare and makeup industry accounting for nearly 40% and 17% of the market share, respectively.^5^ This growth can be attributed to the enormous influence of social media and the evolving role of women in society, which also increased the consumer demand for innovative and high-performance products. As a result, modern cosmetics have complex formulations with many synthetically derived ingredients and additives for stability and other chemical properties.^6^ Although there has been a shift toward more natural substances,^7^ synthetic dyes are preferentially used because of their low production cost, brightness and stability. Despite their widespread use, some of these synthetic dyes have adverse effects on human health.^8^

Owing to potential health risks, regulatory frameworks are needed to ensure the safety and quality of cosmetic products. Cosmetics are considered adulterated if they contain harmful, toxic, decomposed, or filamentous substances. They are also deemed to be adulterated if stored in containers made of poisonous materials or if the containers themselves are contaminated.^9^ India follows a strict, standards-based regulatory approach with a strong emphasis on testing, where violations can lead to penalties ranging from fines to imprisonment.^10^ In the EU, a preventive approach is followed, using positive and negative lists along with a strong emphasis on toxicological safety evaluation, while in the US, manufacturers are responsible for ensuring the safety of cosmetics.^11^

Despite these regulatory measures, cosmetic products may still contain harmful or excessive levels of certain compounds, such as parabens, whitening agents such as hydroquinone, salicylic acid, corticosteroids, and heavy metals such as lead, aluminium, iron, arsenic, cadmium, and formaldehydes, which have been shown to cause well-known side effects, such as leukoderma, irritant dermatitis, increased lifetime cancer risk, reproductive problems, stomach irritation, impaired DNA repair, and organ damage.^12–18^ Nanoparticles such as titanium dioxide have been shown to cause lung inflammation in animals.^19^ Among other components, dyes are also used in cosmetics, which are important because of their toxicity to human health. The majority of azo dyes have been shown to be carcinogenic, toxic and mutagenic, while anthraquinone dyes have shown strong toxic effects. Triarylmethane-based dyes are carcinogenic and severely impact metabolism, whereas xanthene dyes cause changes in the texture of skin because they are rough.^20^ Natural dyes derived from plants, animals and microorganisms are also used in cosmetic formulations that include dyes such as carotenoids, anthocyanins, flavonoids, *etc.*, but these dyes are not durable because they are sensitive to various factors such as pH, temperature, oxidation, metal ions, and light. As these dyes are derived from different species, the dyes show molecular diversity.^21,22^

Lipstick formulations generally consist of waxes, oils, pigments, binding agents, preservatives, emollients, antioxidants and flavouring agents that contribute to product stability, texture, color, spreadability and moisturization properties. Synthetic lipsticks may contain paraffin wax, butyl stearate, oleyl alcohol, parabens, titanium dioxide and synthetic coloring agents. On the other hand, natural-based lipstick formulations may include waxes such as beeswax, plant-derived oils and coloring agents such as cocoa and turmeric.^23^

**Fig. 1:**
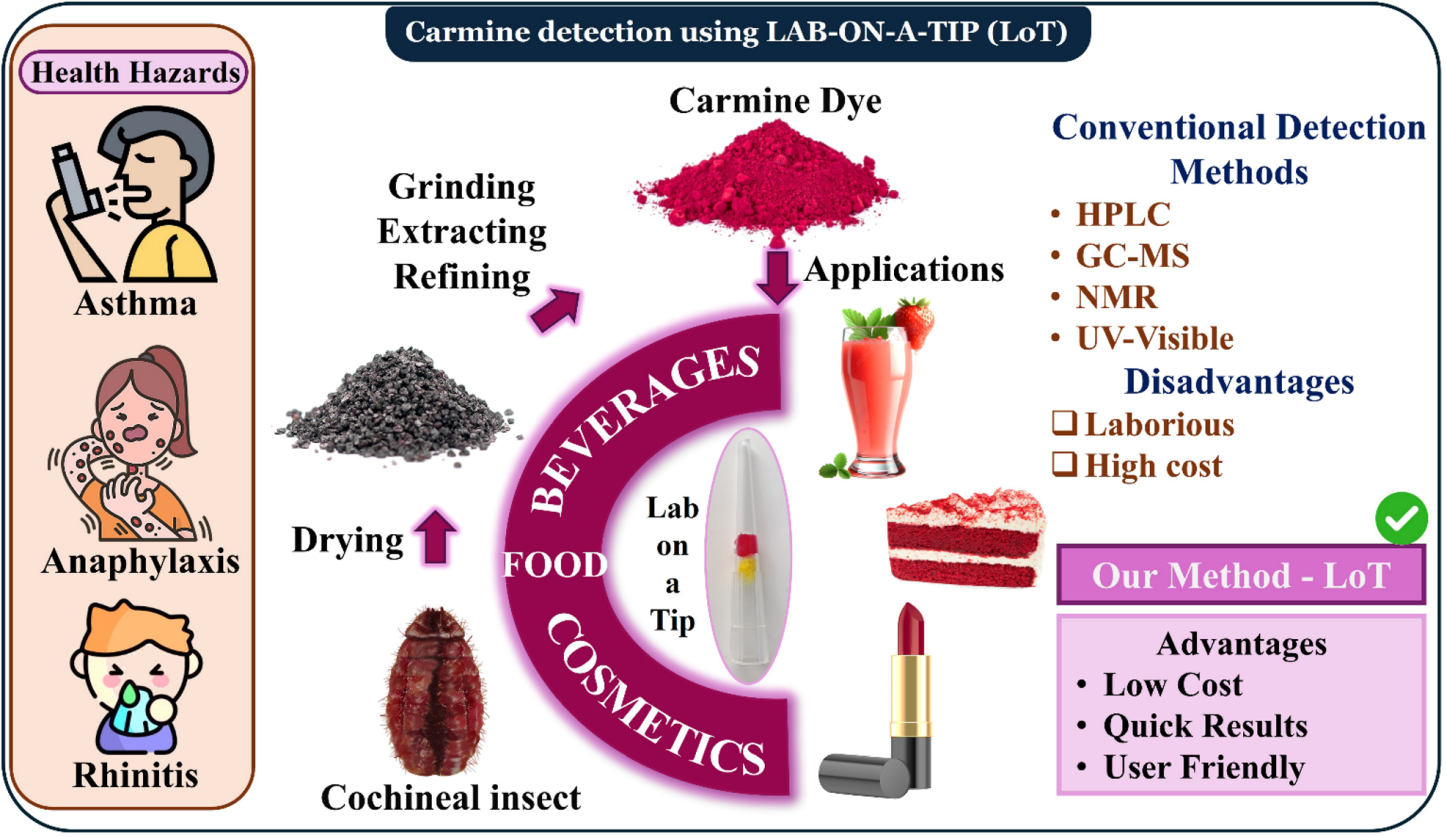
Schematic illustration of the detection of carmine using the lab-on-a-tip system.

Among the various colouring agents used in lipsticks, carmine is widely used as a natural red colorant. The chemical name is hydrated aluminium chelate of carminic acid, and it is also known as natural red 4, E 120, cochineal carmine, C.I. natural red 4.^24^ It is a semi-synthetic red dye derived from the dried bodies of female *Dactylopius coccus*, where the characteristic red colour is attributed to carminic acid. Carmine is widely used in lipsticks, lip balms, blushes, nail polishes and eyeshadows. In addition, carmine is also used as a food colorant in confectionaries, beverages and dairy products.^25,26^ However, it shows considerable batch-to-batch variability, as differences in climate, soil type, moisture and season greatly affect the quality of cochineal plants. Nevertheless, it is among the most stable natural dyes and is highly stable against temperature, light and oxidation.^27^ As carmine is an insect-derived pigment, its detection is highly important for vegetarian and vegan consumers. Additionally, residual insect protein poses potential health risks, as it can cause allergic reactions in severe cases involving anaphylaxis.^28,29^ Hence, rapid detection methods are essential for regulatory authorities. Several conventional analytical methods used for the detection of carmine are based on spectrophotometry, capillary electrophoresis, ultrahigh-performance liquid chromatography, differential pulse polarography, and reversed-phase high-performance liquid chromatography, but these methods are expensive, time consuming, require skilled personnel and are not suitable for rapid onsite detection (Fig. 1).^30–32^

Advances in microfabrication and miniaturized analysis have led to an increase in the number of sophisticated microfluidic devices that integrate multiple processing steps into a single device. This has led to improved device performance because of dominant microscale and nanoscale phenomena that are negligible in macroscale systems.^33–36^ Micropipettes are widely used in laboratories for accurate liquid dispensing, but early developments by researchers such as Zhang and Dejmkova demonstrated the use of micropipette tips as a miniaturized platform for sensing applications. These works provide the foundation for lab-on-a-tip systems by showing how tips can be used as a microenvironment for reaction and detection purposes.^37–39^ Pipette tips are ergonomic and easy to handle and use inexpensive disposable tips that help prevent cross-contamination between samples.^40^ Pipette tips have been used for various other purposes, such as for performing electrochemical measurements,^41^ as miniature columns,^42^ as an electrode body, and as a reference electrode^43^ for colorimetric detection^37^ among other uses, which demonstrates their versatility. The use of Lab-on-a-Tip (LoT) offers several advantages, including a higher surface area-to-volume ratio and the ability to work with smaller sample volumes because of reduced assay dimensions.^33^ Based on these properties, robust, reliable and low-cost devices are increasing in demand in places with limited resources largely because of portability. In this research, we propose the use of micropipette tips for the detection of carmine. The developed system uses the LoT approach by incorporating cotton matrices embedded with reagents in the tip to facilitate controlled chemical reactions. The geometry and design of the device allow for rapid, efficient and onsite detection of carmine in commercial samples. The proposed method also addresses the growing need for the detection of insect-derived dyes, thereby contributing to consumer safety, ethical transparency and regulatory compliance.

## 2 Materials and methods

Pure carmine powder was obtained from Loba Chemie Pvt. Ltd, India. Absorbent cotton and flat cotton were obtained from Prabhat Surgical Cotton Pvt. Ltd and a local cotton dealer in Udupi district, Karnataka, India, respectively. Pipette tips 20–200 µL in volume were obtained from Tarsons Product Ltd, India. Hydrochloric acid (35% w/v extra pure) and ethyl acetate (99.5% v/v extra pure) were obtained from Loba Chemie Pvt Ltd, India. Ferric chloride (Anhydrous) LR was purchased from Molychem India LLP. Ammonia solution (30% extra pure AR) was obtained from SRL, India. A compass, lighter and ink dropper was purchased from a local shop. Whatman® filter paper (grade 1) with a 180 µm thickness and 11 µm pore size was purchased from Whatman®, India. Capillary tubes were obtained from the laboratory material supplier, India.

### 2.1 Sample and reagent preparation

Pure carmine powder was measured and dissolved in 0.2% v/v ammonium hydroxide (NH_4_OH) to prepare a stock solution of 2 mg mL^−1^. Different concentrations of carmine solution (0.05, 0.1, 0.5, 1.0, 1.5 and 2.0 mg mL^−1^) were prepared from the stock solution. 5 N hydrochloric acid (HCl) was prepared by dilution from the concentrated stock of 12 N HCl. Ammonium hydroxide (0.2% v/v) was diluted from the 30% w/v concentrated ammonia solution. A 0.15 M ferric chloride (FeCl_3_) solution was prepared by dissolving 0.243 g of the same solution in 10 mL of distilled water, and it was stored in amber-colored Falcon tubes.

### 2.2 UV-visible spectrophotometric analysis

First, a spectral scan was performed using a BioTek Synergy H1 Microplate Reader to determine the maximum absorption wavelength (*λ*_max_) of the colored complex formed upon reaction of 2 mg mL^−1^ carmine with 5 N HCl and 0.15 M FeCl_3_. Afterward, approximately 330 µL of varying carmine concentrations (0.05, 0.1, 0.5, 1.0, 1.5 and 2 mg mL^−1^) were transferred to 2 mL amber-colored vials and mixed properly with equal volumes of hydrochloric acid and ferric chloride. Measurements were performed in triplicate (*n* = 3) for all the different carmine concentrations, and the absorbance values were measured at 500 nm. A calibration curve was plotted to show the relationship between the mean absorbance ± standard deviation (with the standard deviation represented by error bars) and different concentrations of carmine.

### 2.3 Comparative evaluation of color intensity across different cellulose substrates

The filter paper dots, approximately 4 mm in diameter, were punched using a Kangroo FP-20 punching machine.^44^ To the paper dots, 2 µL of different concentrations of carmine (0.05, 0.1, 0.5, 1.0, 1.5 and 2.0 mg mL^−1^) was applied, followed by the addition of an equal volume of 5 N hydrochloric acid, which converted carmine to carminic acid. After 10 minutes of incubation, 2 µL of ferric chloride was added, and pictures of the different filter paper dots were captured after 15 minutes of incubation. Three trials of the experiment (*n* = 3) were performed for all the carmine concentrations.

The same procedure was followed for the cotton substrates. Absorbent and flat cotton weighing approximately 1.5 ± 0.06 mg was taken and rolled into a small spherical form with a diameter of 4 mm (cotton blobs).^45^ A volume of 15 µL of different carmine concentrations (0.05, 0.1, 0.5, 1.0, 1.5 and 2.0 mg mL^−1^) was added to the different cotton blobs, followed by the addition of an equal volume of 5 N hydrochloric acid. The samples were incubated for 10 minutes for the complete release of carminic acid, as confirmed by the color change from reddish pink to orangish pink. Next, 15 µL of 0.15 M FeCl_3_ was added, and images of different single cotton blobs were captured after 15 minutes of incubation to allow complete formation of the coloured complex. The experiments were conducted in triplicate (*n* = 3) for each carmine concentration. A bar graph was constructed for both paper dots and cotton blobs to show the relationship between the mean grayscale intensity ± standard deviation (with the standard deviation represented by error bars) and different concentrations of carmine.

### 2.4 Bright field microscopy analysis of cotton blobs

A small cotton blob was taken and observed using an inverted bright-field microscope (OX.2053-PLPH, Oxion Inverso Trinocular Microscope, Euromex, Holland), and the images were taken using microscope camera software.^45^

### 2.5 Lab-on-a-tip (LoT) device fabrication

**Fig. 2:**
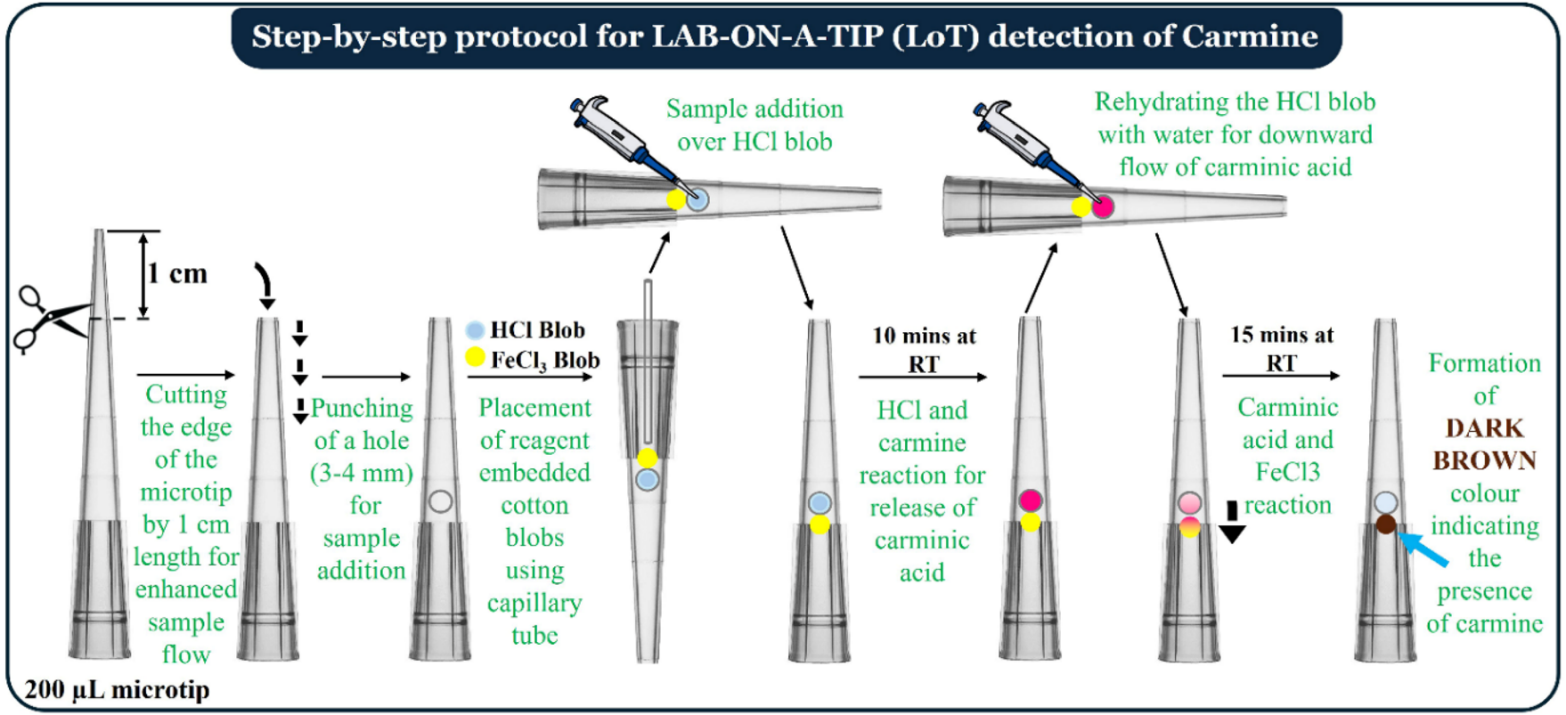
Schematic illustration of LoT, showing fabrication, analyte loading and colorimetric determination of carmine.

A standard 20–200 µL microtip was modified by cutting 1 cm from the distal end of the tip. A metal tip (compass needle) was gently heated, and a small hole was introduced 2 cm from the cut end. Cotton weighing 1.5 ± 0.06 mg was taken and rolled into small spherical forms. Two different cotton blobs were then separately impregnated with 20 µL of 0.15 M FeCl_3_ and 5 N HCl. Cotton blobs impregnated with HCl were dried for 48 hours, whereas FeCl_3_-impregnated cotton blobs were dried overnight at room temperature. After the reagents were completely dried in the cotton blob, the reagent-loaded cotton blobs were slowly added to the modified microtip using capillary tubes (Fig. 2), such that the cotton blob impregnated with HCl was placed adjacent to the hole made on the tip. An FeCl_3_-impregnated cotton blob was added on top of the first blob, ensuring complete contact between the two cotton layers.

### 2.6 Standard analysis of carmine

On the basis of the experiments performed and the results obtained on the cotton blobs previously, standard carmine solutions of different concentrations were evaluated further in the fabricated LoT device. Carmine sample introduction and the addition of water for rehydration were performed through the punched hole for analysis. Twenty microlitres of standard carmine solution of varying concentrations was added slowly to the first cotton blob impregnated with HCl, and the device was incubated for 10 minutes at room temperature while the tip was kept vertical, after which 50 µL of water was added for rehydration and transfer of the solution to the FeCl_3_ blob. The device was further incubated for 15 minutes at room temperature in the vertical position, and the images of color change observed were captured under natural light for further analysis using a smartphone (Oppo A5 Pro 5G, 1× zoom, Auto HDR mode) placed 70 mm from the device. Three trials of the experiment (*n* = 3) were performed, and the images were analysed with the FIJI software.

To analyse the shelf life and evaluate the stability of the stored HCl- and FeCl_3_-impregnated cotton blobs, the same protocol was followed. Three trials of the test (*n* = 3) were performed using standard carmine concentrations (0.05, 0.1, 0.5, 1.0, 1.5, and 2.0 mg mL^−1^). The HCl- and FeCl_3_-impregnated cotton blobs were stored in a closed Petri plate covered with aluminum foil at room temperature for 2 months prior to analysis. Triplicate trials (*n* = 3) were conducted, and the images were analysed with FIJI software.

A bar graph was constructed for fresh and stored LoT devices to show the relationship between the mean grayscale intensity ± standard deviation (with the standard deviation represented by error bars) and different concentrations of carmine. Statistical significance analysis of the color intensities obtained for standard carmine samples in either of the devices was evaluated using one-way analysis of variance (one-way ANOVA) followed by Tukey's multiple comparison test (mean separation method) for the respective plots. The level of significance was considered as *p < 0.05, **p < 0.01, ***p < 0.0005 and ****p < 0.0001.

### 2.7 FT-IR analysis

The functional groups present in the standard carmine, commercial lipstick samples and the reagents in the respective reactions were analysed by an FTIR spectrometer (Bruker Alpha II, India) in the ATR mode (in the range of 400–4000 cm^−1^).

### 2.8 Commercial sample analysis

Commercial lipstick samples were procured from local cosmetics stores in Manipal, Karnataka, India. To identify a suitable solvent to extract carmine from solid lipstick, a solubility test was performed using various solvents, such as ammonium hydroxide, ethyl acetate, ethanol, acetone, sodium bicarbonate, a mixture of acetone and ethanol (50 : 50, v/v) and a mixture of ethanol and ethyl acetate (50 : 50, v/v).

A small amount of lipstick (3 ± 0.2 mg) was scraped and dipped in 50 µL of ethyl acetate to obtain a suspended solution. Twenty microliters of this extract was added to the fabricated lab-on-a-tip device. After 10 minutes, 20 µL of water was added to facilitate rehydration, and images were captured after 15 minutes of incubation. The rehydration volume used was 20 µL to avoid dilution of the carmine concentration present in the heterogeneous sample matrices. The images were further analysed, and different intensities were observed using FIJI software. To evaluate the colorimetric response of the LoT device in the detection of carmine, a known carmine-free lipstick sample was analysed using the same protocol for comparison.

To evaluate solvent suitability, the same protocol was followed in which 3 ± 0.2 mg of lipstick was dipped in 50 µL of 0.2% NH_4_OH. Lipstick samples prepared in both 0.2% NH_4_OH and ethyl acetate were further treated with HCl and FeCl_3_ and analysed using UV-vis spectroscopy, where the same protocol used for the standard carmine solution prepared in NH_4_OH was followed.

LoT device-based analysis of different commercial lipstick samples suspended in EtOAc and NH_4_OH was performed, and a bar graph showing the grayscale intensity values obtained for the samples along with the control was constructed.

#### 2.8.1 Recovery analysis using lipstick sample spiked with carmine

Commercial carmine-free lipstick sample was melted at 100 °C to obtain 1 mL of lipstick matrix suspension. 2 mg of pure carmine was added to the suspension and mixed properly using a glass capillary tube. The mixture was allowed to solidify at room temperature, and the further analysis of the spiked lipstick sample was performed using the same protocol as commercial lipstick sample through UV-vis spectrophotometry and LoT device. The recovery of carmine from spiked lipstick sample was calculated by comparing with standard carmine sample.

The green metric score of the proposed methodology was calculated using AGREE (Analytical GREennEss Calculator) software.^46,47^

## 3 Results and discussion

Carmine is widely used as a colorant in cosmetics such as lipsticks, blushes and eyeshadows as well as in food products such as yogurt, jams, ice cream and even soft drinks.^25,48^ However, its presence in food, beverages and cosmetics has caused IgE-mediated hypersensitivity in many individuals, including those with angioedema, urticaria, contact dermatitis, systemic anaphylaxis and even asthma.^25,31,49–51^ To detect carmine in cosmetics, we developed a simple, user-friendly lab-on-a-tip system.

### 3.1 UV-visible spectrophotometric analysis

**Fig. 3:**
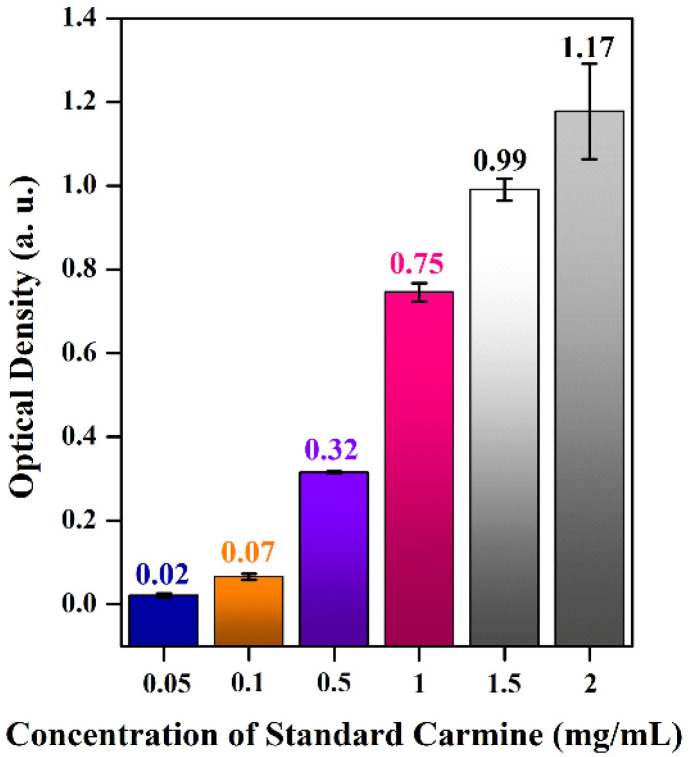
Calibration curve showing the relationship between absorbance and different carmine concentrations, with error bars representing standard deviations.

First, we performed bulk analysis to study the interactions between carmine, HCl and FeCl_3_ across a wide range of concentrations (SI Fig. S1). This helped us observe brown complex formation, with its color intensity increasing with increasing carmine concentration. After analysing the data and plotting a calibration curve, we observed a positive correlation between the absorbance and concentration, confirming that the reaction is suitable for quantitative analysis (Fig. 3). The LOD and LOQ of the bulk method was calculated as per the standard IUPAC approach (LOD = 3.3 *σ*/*S* and LOQ = 10 *σ*/*S*), which was determined to be 0.35 mg mL^−1^ and 1.05 mg mL^−1^, respectively (SI Fig. S4 and SI Table S2). Since the scatter in the calibration data was found to be relatively high, the mathematically calculated LOD and LOQ values were inflated. Hence, accurate LOD and LOQ values could not be determined. The values were then experimentally verified using replicate absorbance measurements at the lower concentration levels and confirmed by observing the visual colour response. 0.05 mg mL^−1^ and 0.1 mg mL^−1^ carmine exhibited relative standard deviation (RSD) of 24.74% (∼25%) and 8.66%, respectively, whereas 0.5 mg mL^−1^ carmine showed RSD of 1.84%. Therefore, 0.5 mg mL^−1^ was considered the practical LOD and LOQ of the method.

### 3.2 Comparative evaluation of colour intensity across different cellulose substrates

Point-of-care devices mostly use cellulose substrates such as paper and cotton. To identify a suitable substrate for our present work, we decided to first compare cotton blobs and filter paper. The 4 mm paper dots were selected for meaningful comparison between the cotton and the filter paper, as the cotton blobs were measured to have the same diameter. As cotton has an increased absorption capacity, high porosity, and a 3-dimensional structure, the total volume was optimized to 45 µL to achieve uniform reagent interaction and saturation. In contrast, paper dots are planar and can absorb a lower volume than cotton can absorb; hence, the total volume is only 6 µL.

**Fig. 4:**
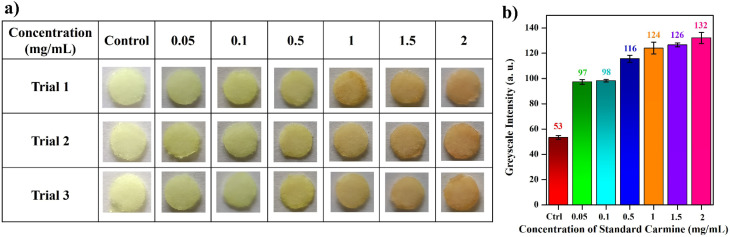
(a) Visual representation of filter paper dots subjected to different concentrations of carmine. (b) Semiquantitative colorimetric analysis of filter paper dots using FIJI software, indicating a positive correlation between absorbance and concentrations, with error bars representing standard deviations.

**Fig. 5:**
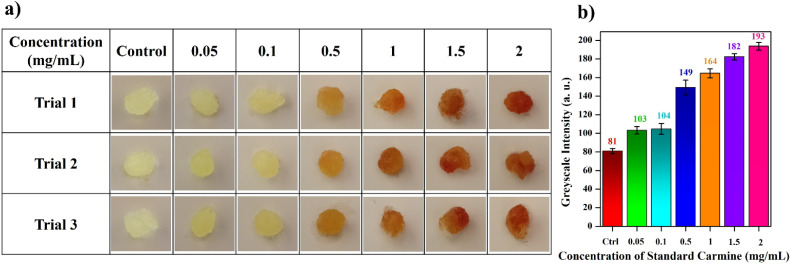
(a) Visual representation of cotton blobs subjected to different concentrations of carmine. (b) Semiquantitative colorimetric analysis of cotton blobs using FIJI software, indicating a positive correlation between absorbance and concentrations, with error bars representing standard deviations.

We observed that the color intensity of the cotton blobs qualitatively increased. Furthermore, it was validated through quantitative image analysis using FIJI software, which confirmed that compared with filter paper, cotton can be better distinguished at lower concentrations (Fig. 4, 5 and SI Fig. S6). The enhanced performance can be attributed to the intrinsic properties of cotton, including its fibrillar and crystalline structure, high porosity and greater absorption capacity, which increase its dye uptake and retention. Additionally, the three-dimensional intertwined fibrous network of cotton provides a large surface area for interaction, formation and visualization of the chromogenic complex formed.^52,53^ Therefore, a potential alternative for such point-of-care applications would be cotton. Additionally, among the two types of cotton material used (absorbent and flat cotton), absorbent cotton blobs exhibited enhanced colour response compared to flat cotton material (Fig. 5 and SI Fig. S6).

**Fig. 6:**
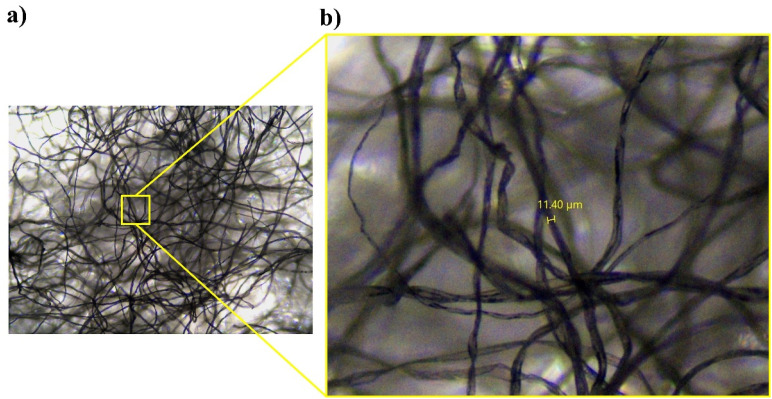
(a) Fibrous 3D network of cotton blob captured using Brightfield microscope at 40× magnification. (b) The fibre width was measured to be approximately 11.40 µm.

To assess and further validate the enhanced performance of the cotton blobs, we performed a microscopic analysis. Upon analysis, we observed a dense, intertwined network of fibres, and the width of the fibres was calculated to be 11.40 µm (Fig. 6). The fibrillar structure creates a highly porous matrix that has an enhanced holding capacity and creates efficient capillary action. The 3D interconnected fibrous network also has increased surface area, which enables proper formation and visualization of chromogenic complexes. Compared with filter paper, which has a more planar structure, these structural properties of cotton enhanced the color intensity and improved the sensitivity at even lower concentrations.

### 3.3 Standard analysis of carmine in LoT devices

**Fig. 7:**
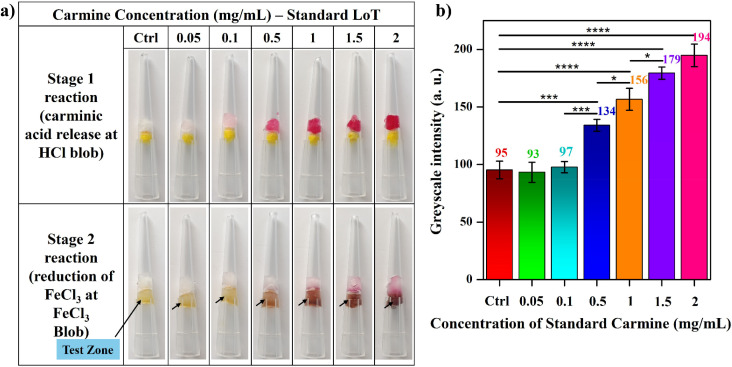
(a) Concentration-dependent colorimetric response of the Lab-on-a-Tip (LoT) device for carmine detection. Stage 1 shows the release of carminic acid in the HCl-treated layer, while Stage 2 illustrates the formation of a brown chromogenic complex upon interaction with FeCl_3_. Increasing carmine concentration (0–2 mg mL^−1^) results in a progressive intensification of colour, demonstrating effective analyte diffusion and reaction across the layered system. (b) Semiquantitative analysis of carmine detection using greyscale intensity measurements. Images were analysed using FIJI software, showing a clear increase in greyscale intensity with rising carmine concentration (0–2 mg mL^−1^), confirming the sensitivity and reproducibility of the LoT device. Mean values (*n* = 3) are presented with error bars representing standard deviation. Statistical significance among the intensities obtained with different concentrations of carmine was evaluated using one-way analysis of variance (one-way ANOVA) followed by Tukey's multiple comparison test. Level of significance *p < 0.05, **p < 0.01, ***p < 0.0005, ****p < 0.0001, and no symbol = insignificant.

Standard carmine solutions were used to test the accuracy and reproducibility of the fabricated lab-on-a-tip device. During the incubation, no movement of carmine from the first blob to the second blob was observed. A wide range of carmine concentrations was used, and a concentration-dependent change in the brown chromogenic complex was observed. The images were analysed using FIJI, which revealed an increasing trend in color intensity with increasing concentration (Fig. 7). The LOD of the LoT device was experimentally determined to be 0.5 mg mL^−1^ on the basis of the statistical analysis performed using one-way analysis of variance (one-way ANOVA) followed by Tukey's multiple comparison test.

#### 3.3.1 Shelf-life analysis

**Fig. 8:**
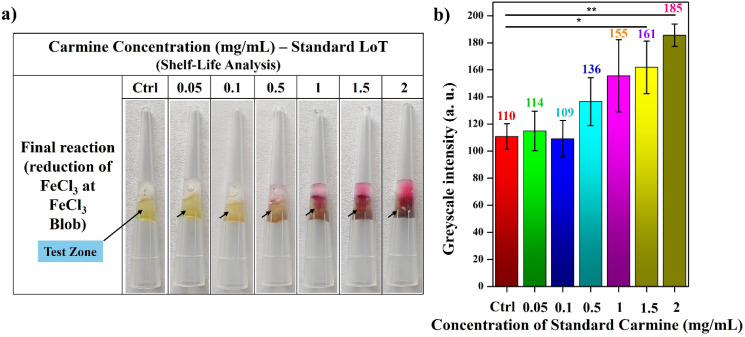
(a) Shelf-life analysis of LoT device using standard carmine (b) greyscale intensity values analysed using FIJI software, showing a clear increase in greyscale intensity with rising carmine concentration (0–2 mg mL^−1^). Mean values (*n* = 3) are presented with error bars representing standard deviation. Statistical significance among the intensities obtained with different concentrations of carmine was evaluated using one-way analysis of variance (one-way ANOVA) followed by Tukey's multiple comparison test. Level of significance *p < 0.05, **p < 0.01 and no symbol = insignificant.

Compared with the freshly prepared devices, the stored devices retained comparable colorimetric response characteristics, with a slight reduction in mean intensity and significant changes in intensity for the 1.5 and 2 mg mL^−1^ treatments with respect to the control, although a slight reduction in the yellow colouration of the FeCl_3_-impregnated cotton blobs was visually observed during prolonged storage (Fig. 8).

#### 3.3.2 Relative standard deviation (% RSD) analysis

The precision of the developed LoT-sensing platform was evaluated through intraday and interday reproducibility studies using triplicate measurements over a concentration range of 0.05–2 mg mL^−1^ (SI Table S5). The obtained RSD values demonstrated acceptable reproducibility, particularly at moderate and high analyte concentrations (interday RSD % of 0.92 and 0.47 for 0.5 and 1 mg mL^−1^, respectively). The higher RSD values observed at lower concentrations (interday RSD % of 14.12 for 0.05 mg mL^−1^) may be attributed to reduced signal intensity and inherent experimental variability near the detection limit.

### 3.4 Commercial sample analysis

In the present study, the calibration standards were prepared in 0.2% v/v NH_4_OH to ensure efficient dissolution of pure carmine. Carmine is a highly polar compound and exhibited poor solubility in ethyl acetate during preliminary experiments owing to the nonprotic nature of the solvent. In contrast, the use of ammonium hydroxide as a protic solvent provided effective solubilization and stabilization of carmine in solution form, thereby enabling the preparation of reliable calibration standards (SI Fig. S2).

Ethyl acetate was employed for the extraction of carmine from the commercial lipstick matrix because of its ability to efficiently dissolve the waxy and hydrophobic components of the lipstick. The extraction step converts the heterogeneous semisolid cosmetic matrix into a workable liquid suspension, improving the accessibility of embedded dye molecules to hydrochloric acid and ferric chloride during color development. In contrast, ammonium hydroxide was not suitable for dissolving the highly hydrophobic matrix (SI Fig. S3). Moreover, ethyl acetate is available in common household products, such as nail polish remover, and is easily accessible to the user for everyday use.

**Fig. 9:**
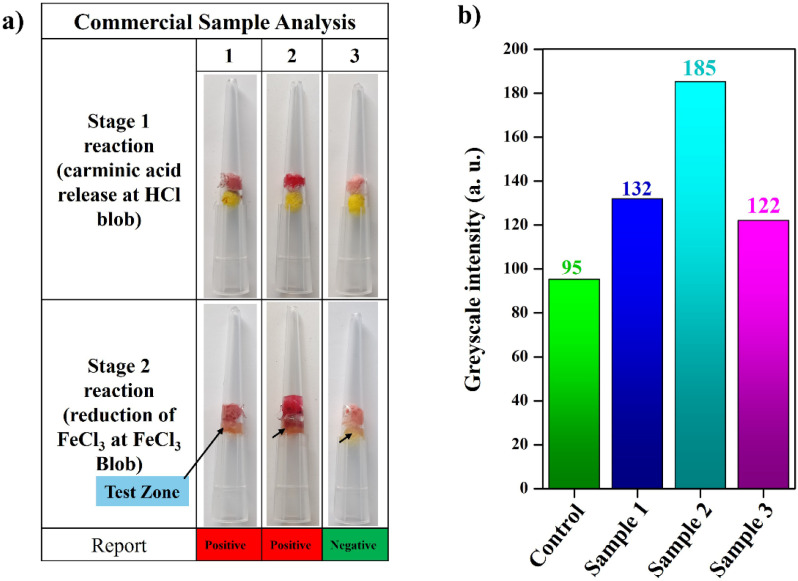
(a) Qualitative detection of carmine in commercially available lipstick samples dissolved in ethyl acetate and analysed using the LoT device. Among the tested samples, one lipstick sample showed no visible colour change (negative control), whereas other lipstick samples exhibited distinct brown colouration (positive), indicating the presence of carmine. Variation in colour intensity suggests differences in carmine content across brands. (b) Semiquantitative grayscale intensity analysis of commercial samples using the LoT device. Grayscale intensity values obtained from FIJI analysis show higher intensity for lipstick samples compared to the food sample, confirming the presence and relative variation of carmine in cosmetic products.

To assess the practical applicability of such a robust and zero-cost LoT platform, we tested our device on commercially available carmine-containing and carmine-free lipstick samples. Two lipstick samples (suspended in EtOAc) tested revealed a distinct brown color, indicating the presence of carmine, whereas one sample did not exhibit this color (Fig. 9). Variation in brown color intensity was observed among different brands, suggesting a difference in the amount of carmine used (in the range of 0.5–2 mg mL^−1^), which was semiquantitatively distinguished in our device and almost comparable to the bulk detection data (SI Table S3). The developed LoT device-based sensing technique achieved a green score of 0.74, proving it to be a green method (SI Fig. S7).

To determine the incompatibility of NH_4_OH as an extraction solvent for commercial sample analysis, LoT and UV-vis spectrophotometric-based measurements were performed, and the results were compared with EtOAc solvent data (Fig. 9 and SI Fig. S5). Negligible color development was observed in the LoT devices for the NH_4_OH-suspended commercial samples. Similarly, low absorbance values were recorded for the samples analysed using UV-vis spectrophotometry. Additionally, the % color response based on grayscale intensity values with respect to the control measured for both datasets confirmed the extraction efficiency of carmine with EtOAc (SI Table S4). The analysis performed for the lipstick sample spiked with 2 mg mL^−1^ carmine using UV-visible spectrophotometry and LoT device revealed a recovery percentage of 80.7% and 89.4%, respectively (SI Table S6 and SI Fig. S8).

Ferric chloride is a relatively nonspecific reagent and can react with phenolic compounds, polyphenols, tannins, flavonoids and chelating agents present in cosmetic and food matrices to form colored complexes. However, the colorimetric response produced with FeCl_3_ varies depending on the analyte. Previous studies have reported that phenolic compounds, tannins and flavonoids produce dark green, deep blue, bluish black, bluish green, violet, greenish black, blackish red, reddish brown or green–blue colouration upon reaction with FeCl_3_.^54,55^ Methylparaben, a commonly used synthetic preservative in lipstick and cosmetics, forms a violet-colored complex with FeCl_3_.^56^ In comparison, the dark-brown colouration observed for the carminic acid–FeCl_3_ reaction has been less commonly reported, except for certain constituents present in beetroot root extract and aloe leaves.^54^ Since plant-derived materials present in natural lipstick may contain phenolic compounds capable of reacting with ferric chloride, interference from such compounds present in natural lipstick cannot be completely ruled out. Moreover, the exact composition of the lipstick is a trade secret.

### 3.5 FT-IR analysis

**Fig. 10:**
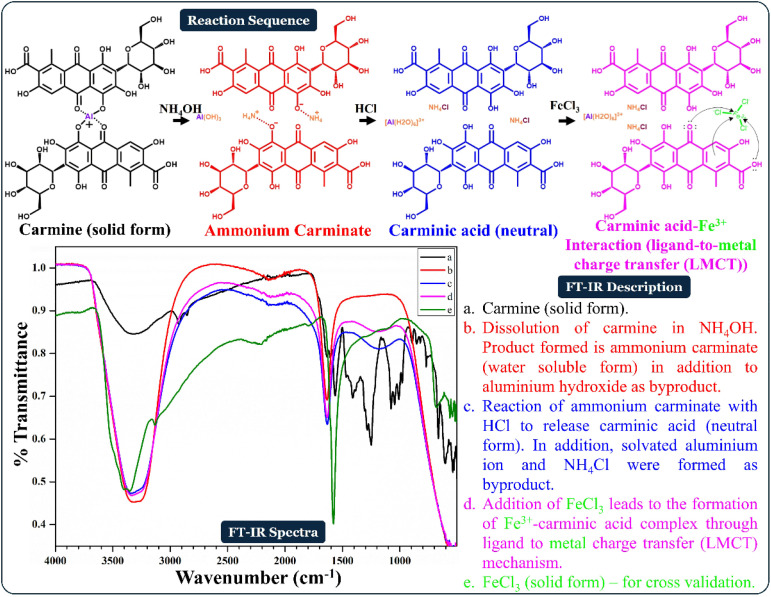
FT-IR spectra of (a) carmine (solid), (b) carmine in NH_4_OH, (c) reaction of b with HCl, (d) reaction of c with FeCl_3._

**Fig. 11:**
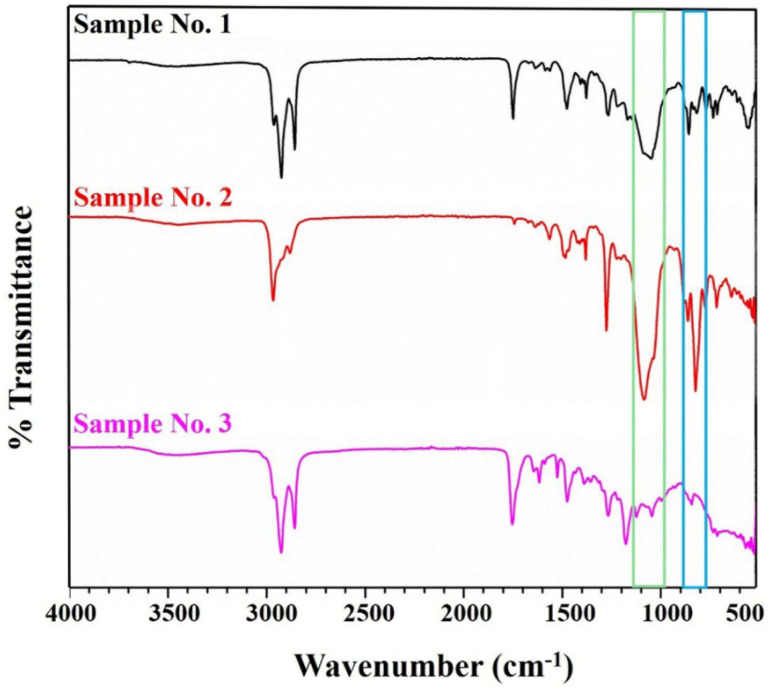
FT-IR spectra of commercial lipstick samples.

To validate the presence of carmine in the lipstick samples, we carried out FT-IR spectral analysis for both commercial and standard carmine samples, as depicted in Fig. 10 and 11. FT-IR spectra of carmine (spectra a) confirm the presence of essential aluminum–oxygen coordination bonds and broad O–H stretching bands at 600–500 cm^−1^ and 3400 cm^−1^, respectively. Upon NH_4_OH treatment (spectra b), the transmittance intensity drastically increased and was broad at approximately 3400 cm^−1^ (OH stretching). This occurred because of the intermolecular hydrogen bonding between the ammonium ion and phenolic oxygen atom of carmine. The deprotonation and disruption of the Al–O complex led to the formation of a stable water-soluble ammonium carrier complex. On the other hand, aluminum ions react with the hydroxyl moiety of NH_4_OH and form stable ammonium hydroxide species. Acidification (spectra c) of ammonium carminate using 5 N HCl regenerates carminic acid, which was confirmed by the absence of Al–O/NH_4_^+^ bands and a C=O stretch at approximately 1635–1640 cm^−1^ (slightly decreased because of hydrogen bonding). The mode of interaction between the released carminic acid and FeCl_3_ (spectra d) was confirmed by a subtle shift in the C=O and C–O bands, indicating that Fe^3+^ interactions increased *via* ligand-to-metal charge transfer (LMCT) and not by a coordination mechanism.

Furthermore, FT-IR analysis was performed on three commercial lipstick samples to validate the presence of carmine by comparison with standard carmine samples. In samples 1 and 2, a characteristic aluminum–oxygen (Al–O) vibrational band was observed at 700 cm^−1^, similar to the standard carmine spectrum, confirming the presence of the aluminum–carminic acid complex. In addition, the phenolic C–O stretching band observed in the region of 1050–1070 cm^−1^ was clearly exhibited in samples 1 and 2. However, both the Al–O band and the phenolic C–O stretching band were absent in sample 3. The absence of these characteristic spectral features confirmed that sample 3 did not contain carmine pigment. Therefore, the comparative FT-IR spectral analysis of commercial lipstick samples with standard carmine spectra clearly demonstrated the presence of carmine in samples 1 and 2 but confirmed its absence in sample 3 (carmine-free). Brown color formation is attributed to electron transfer from oxygen atoms and the π-system of the ligand to vacant d-orbitals of Fe^3+^ (Table 1).

**Table 1:** FT-IR assignments (comparative)

Functional group/vibration in cm^−1^	Carmine (solid form)	Carmine in NH_4_OH	Reaction of carmine in NH_4_OH with HCl to release carminic acid	Reaction between FeCl_3_ and carminic acid
O–H stretching	∼3400 (broad)	∼3400 (very broad)	∼3400 (less broad than^b^)	3400 (broad)
C–H stretching	2920–2850	—	—	—
C=O (anthraquinone)	1600–1620	1620	1630	1625
Aromatic C=C	1560 (sharp)	—	—	—
C–O stretching	1280–1220	1200–1100 (weak)	1200–1100	1200–1100
Al–O	600–500	—	—	—

## 4 Conclusion and future perspective

In this work, we used a fabricated lab-on-a-tip device with a two-step colorimetric detection process that confirms the presence of carmine in cosmetic samples, with a limit of detection of 0.1 mg mL^−1^, which was determined experimentally. The system clearly demonstrated concentration-dependent responses, as supported by spectrophotometric and image-based analyses, confirming its reliability and reproducibility. This comparative study established cotton as a superior substrate over filter paper because of its enhanced absorption capacity and structural properties, leading to improved sensitivity. The successful detection of carmine in commercial cosmetics highlights the practical applicability of the device. The high-cost effectiveness (USD $ 0.0055; SI Table S1), portability and ease of operation of the device make it a promising alternative to conventional laboratory-based techniques, particularly for rapid screening and onsite analysis. However, this device is semiquantitative and subjective in nature, hence restricting its usage as a preliminary sensing platform. Although the device is user friendly, minimal training of the users may be required to facilitate proper operation and accurate analysis. Additionally, there is a need to increase the shelf life of the reagent-loaded cotton blobs, owing to the reproducibility issue observed with commercial lipstick matrix analysis. In the future, direct extraction of the lipstick matrix can be explored using a 3D-printed LoT device, with a focus on improving the sensitivity and shelf life under different storage conditions. Integrating smartphone-based quantification can enable real-time automated analysis, with further expansion of the platform for the detection of other dyes or contaminants in food and cosmetics products. This approach has strong potential for development into a versatile onsite detection tool. Furthermore, the FeCl_3_-based colorimetric approach may have limited selectivity for carminic acid in complex matrices. In future studies, we intend to explore more selective chemosensors for improved specificity toward analyte detection.

## Author contributions

BS: conceptualization, methodology, validation, formal analysis, investigation, data curation, writing – original draft; OD: formal analysis, investigation, data curation, writing – original manuscript; SD: methodology, writing – original manuscript; AP: conceptualization, methodology, validation, formal analysis, investigation, data curation, writing – original draft; LP: formal analysis, investigation; HM: formal analysis, writing – review and editing; NKM – conceptualization, methodology, formal analysis, resources, data curation, writing – review and editing, supervision, project administration and funding acquisition.

## Conflicts of interest

There are no conflicts to declare.

## Supplementary Material

RA-OLF-D6RA03072F-s001

## Data Availability

The data supporting this article have been included in the main manuscript and in the supplementary information (SI). Supplementary information is available. See DOI: https://doi.org/10.1039/d6ra03072f.
