# Supplementary material for: Lab-on-a-tip platform with cotton matrices for colorimetric detection of carmine in lipsticks
Source: RSC Adv. 2026 Jul 3;16(35):35633–45. doi: 10.1039/d6ra03072f (PMC13329852; doi:10.1039/d6ra03072f)
Supplement: RA-016-D6RA03072F-s001 [file RA-016-D6RA03072F-s001.pdf]

Supplementary Information (SI) for RSC Advances.
This journal is © The Royal Society of Chemistry 2026

Lab-on-a-Tip Platform with Cotton Matrices for Colorimetric Detection of Carmine in Lipsticks

Balachandar Sundarrajan[a,±], Oyessi Dutta[a,±], Sohini Dutta[a], Anusha Prabhu[a], Lokitha Paduvetnaya[b], Harishkumar Madhyastha[c] & Naresh Kumar Mani[a,*]

Supplementary Table 1: Cost analysis of the Lab-on-a-tip (LoT) device for Carmine detection

**Material stock**		**LoT Device**	
**Particulars**	**Price (INR)**	**Particulars**	**Price (INR)**
Hydrochloric acid, 2.5 L	578	20 µL of 5 N Hydrochloric acid	0.002
Ferric chloride, 500 g	318.6	20 µL of 0.15 M Ferric chloride,	0.0003
Micropipette Tips, 1 bag	505.512	Micropipette Tips, 1 tip	0.51
Absorbent cotton roll, 500 g	252	4 mg of Absorbent cotton roll	0.002
**Grand Total**	1654.11 6	**Grand Total**	0.5143
Total Cost of one Lab-on-a-Tip Device is **₹ 0.5143 INR (USD $ 0.0055)**<br><br>Additional cost for fabrication of the Lab-on-a-Tip (LoT) device involving per day labour cost for a semiskilled worker is approximately ₹ 900 INR or USD 9.38 ~ USD 10 (The cost in terms of equipment which might be incurred in the production of materials has been excluded owing to the direct use of commercially available microtips and absorbent cotton)			

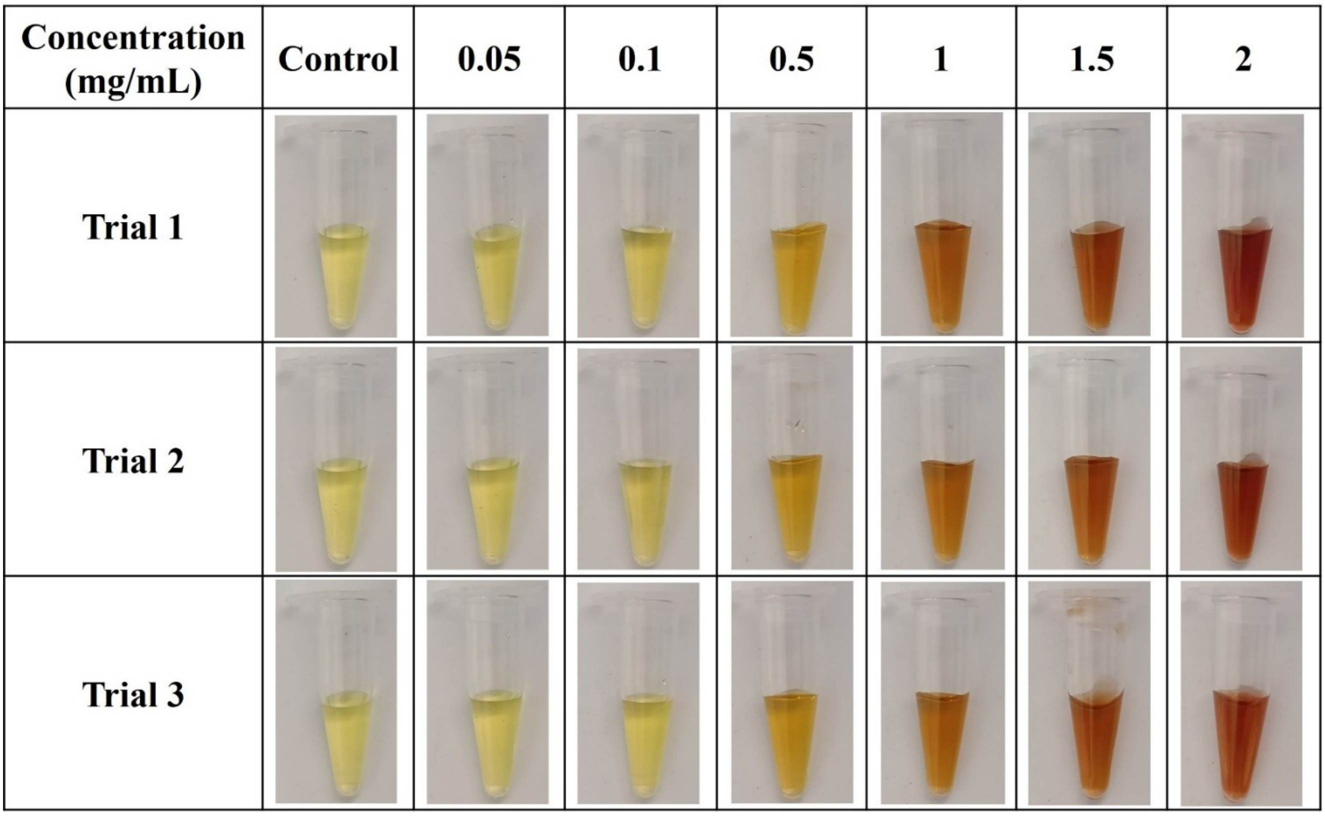

Supplementary Fig. 1: Visual representation of bulk analysis of different concentrations of carmine (0.05, 0.1, 0.5, 1.0, 1.5, 2.0 mg/mL)

Tool Optimization

In the laboratory setting, an analyte can be added to the device using a micropipette; however, this equipment is not easily available to consumers. Therefore, commonly available tools such as DispoVan syringes and 6 mL Pasteur pipettes were tested for volume optimization. For the syringe, 50 µL corresponds to approximately 3 drops, and 1 corresponds to 20 µL. Similarly, for the Pasteur pipette, 1 drop is equivalent to 50 µL, and half of a drop corresponds to 20 µL. This tool optimization demonstrates that the device is user friendly and that carmine can be tested using simple, easily accessible tools.

Solubility of pure carmine powder in different solvents

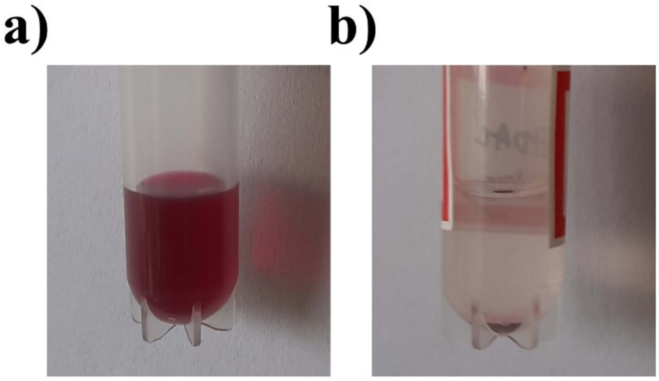

Supplementary Fig. 2: a) Solubility of pure carmine powder in 0.2% NH_4OH showing complete dissolution. b) Solubility of pure carmine in ethyl acetate showing poor solubility and precipitation

Solubility Analysis of Commercial Lipstick Samples

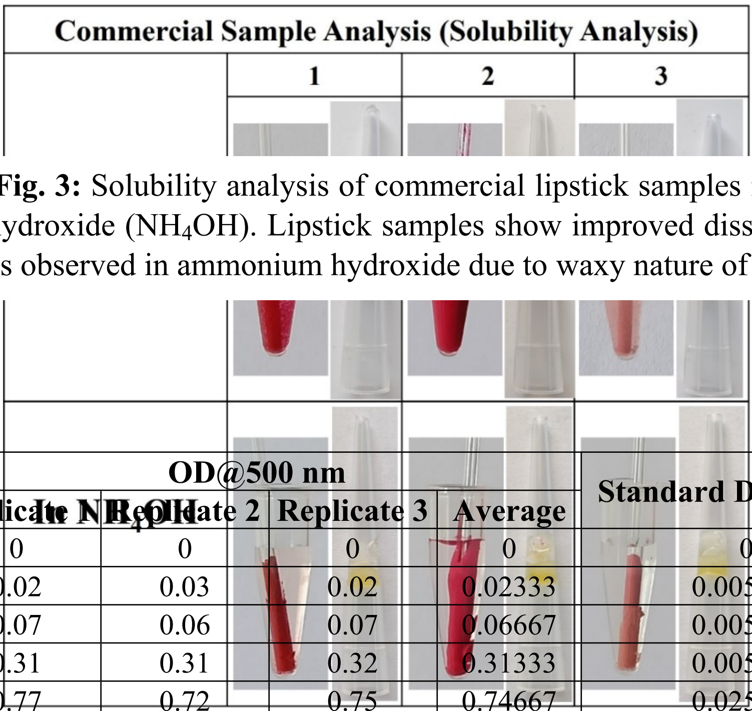

Supplementary Fig. 3: Solubility analysis of commercial lipstick samples in ethyl acetate (EtoAc) and ammonium hydroxide (NH_4OH). Lipstick samples show improved dissolution in ethyl acetate. Poor dissolution is observed in ammonium hydroxide due to waxy nature of lipstick matrix.

Determination of the LOD and carmine concentration in commercial samples using a standard calibration curve

Concentration (mg/mL)	OD@500 nm				Standard Deviation	%RSD
	Replicate 1	Replicate 2	Replicate 3	Average		
0.00 (Control)	0	0	0	0	0	0
0.05	0.02	0.03	0.02	0.02333	0.005774	24.74
0.10	0.07	0.06	0.07	0.06667	0.005774	8.66
0.50	0.31	0.31	0.32	0.31333	0.005774	1.84
1.00	0.77	0.72	0.75	0.74667	0.025166	3.37
1.50	0.98	1.02	0.98	0.99333	0.023094	2.32
2.00	1.05	1.27	1.22	1.18	0.115326	9.77

Supplementary Table 2: Absorbance values obtained at various carmine concentrations.

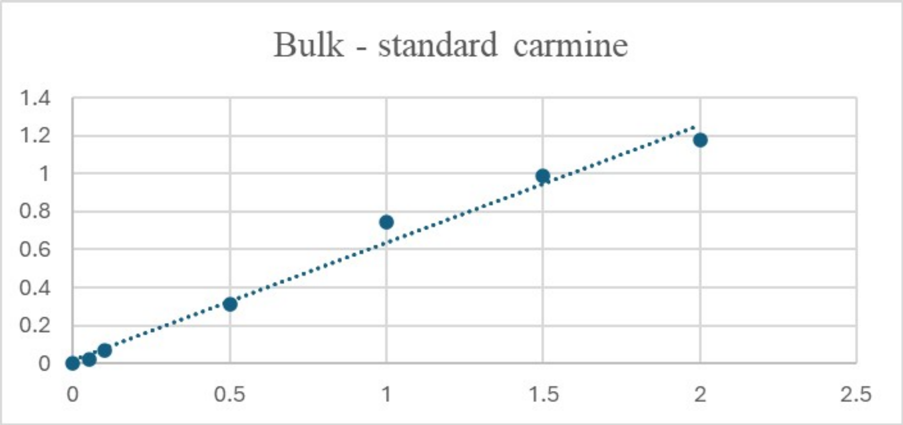

Supplementary Fig. 4: Calibration curve showing relationship between carmine concentrations and absorbance values using UV–vis spectrophotometric analysis.

Slope (S) = 0.622021868

Intercept = 0.01713153

R^2 = 0.985427247

Standard error of y estimate (σ)= 0.065276823

LOD = (3.3 x σ)/S

LOD = (3.3 x 0.065276823)/0.622021868

LOD = 0.346311803 mg/mL

LOQ = (10 x σ)/S

LOQ = (10 x 0.065276823)/0.622021868

LOQ = 1.049429707 mg/mL

Since the scatter in the calibration data was found to be relatively high, the mathematically calculated LOD and LOQ values were inflated. Hence, accurate LOD and LOQ values could not

be determined. The values were then experimentally verified using replicate absorbance measurements at the lower concentration levels and confirmed by observing the visual colour response. 0.05 mg/mL and 0.1 mg/mL carmine exhibited relative standard deviation (RSD) of 24.74% (~ 25%) and 8.66%, respectively, whereas 0.5 mg/mL carmine showed RSD of 1.84%. Therefore, 0.5 mg/mL was considered the practical LOD and LOQ of the method.

Supplementary Table 3: Quantification of carmine concentrations in commercial lipstick samples based on the calibration plot equation (Supplementary Fig. 4)

Name	OD @ 500 nm (y)	Slope	Intercept	Conc.(x)
Control	0	0.6211	0.0166	0
Sample 1	1.898			3.029142
Sample 2	0.94967			1.502286
Sample 3	0.29267			0.444486

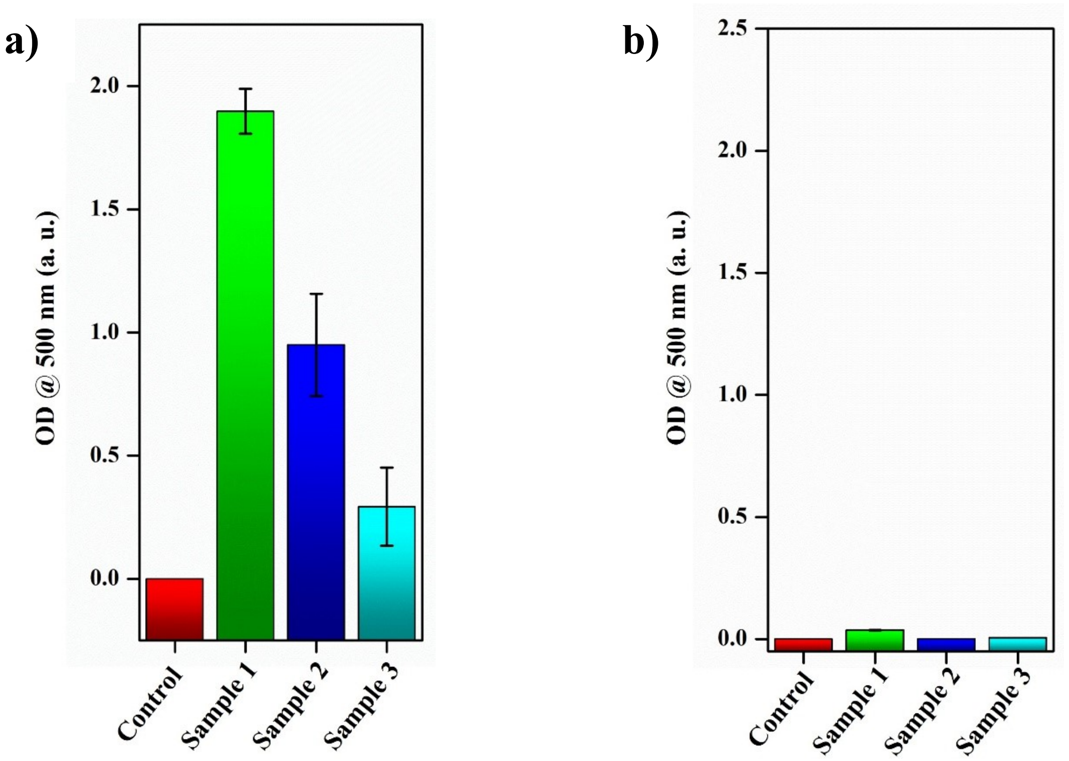

Supplementary Fig. 5: OD@500 nm for commercial lipstick matrix extracted with a) Ethyl acetate b) Ammonium hydroxide

Supplementary Table 4: % color response obtained with commercial lipstick samples extracted with EtoAc and NH_4OH

Sample	Lipstick matrix extracted using EtOAc		Lipstick matrix extracted using NH_4OH	
	**Greyscale intensity obtained in LoT device**	**% colour response compared to control = ((Intensity of sample – Intensity of control)/Intensity of control)*100**	**Greyscale intensity obtained in LoT device**	**% colour response compared to control = ((Intensity of sample – Intensity of control)/Intensity of control)*100**
**Control**	95.278	0	95.278	0
**Sample 1**	132.002	38.54	95.1567	0
**Sample 2**	185.274	94.46	97.898	2.75
**Sample 3**	122.083	28.13	97.3837	2.21

Supplementary Table 5: Relative standard deviation (RSD%) analysis of the developed LoT device

Input Values

Concentration (mg/mL)	Day 1 Mean (μ)	Day 1 SD (σ)	Day 60 Mean (μ)	Day 60 SD (σ)
Control	95.278	7.70185	110.7763	9.40725
0.05	93.24133	8.69628	114.9033	14.67039
0.1	97.73833	4.86281	109.1277	13.58779
0.5	134.109	5.14768	136.5933	17.69551
1	156.653	9.58474	155.626	26.72275
1.5	179.45333	5.28957	161.9347	19.39138
2	194.89867	9.78893	185.6117	8.21331

RSD % = Standard Deviation (σ)/Mean (μ) x 100

Day 1 Intraday RSD (%)

Concentration (mg/mL)	RSD (%)
Control	8.08
0.05	9.33
0.1	4.98
0.5	[illegible]

Day 60 Intraday RSD (%)

Concentration (mg/mL)	RSD (%)
Control	8.49
0.05	12.77
0.1	12.45
0.5	[illegible]

Interday RSD (%)

Concentration (mg/mL)	RSD (%)
Control	10.77
0.05	14.12
0.1	7.73
0.5	0.92
1	0.47
1.5	7.10
2	3.47

The precision of the developed LoT sensing platform was evaluated through intraday and interday reproducibility studies using triplicate measurements over a concentration range of 0.05–2 mg/mL. The obtained RSD values demonstrated acceptable reproducibility, particularly at moderate and high analyte concentrations. The higher RSD values observed at lower concentrations may be attributed to reduced signal intensity and inherent experimental variability near the detection limit.

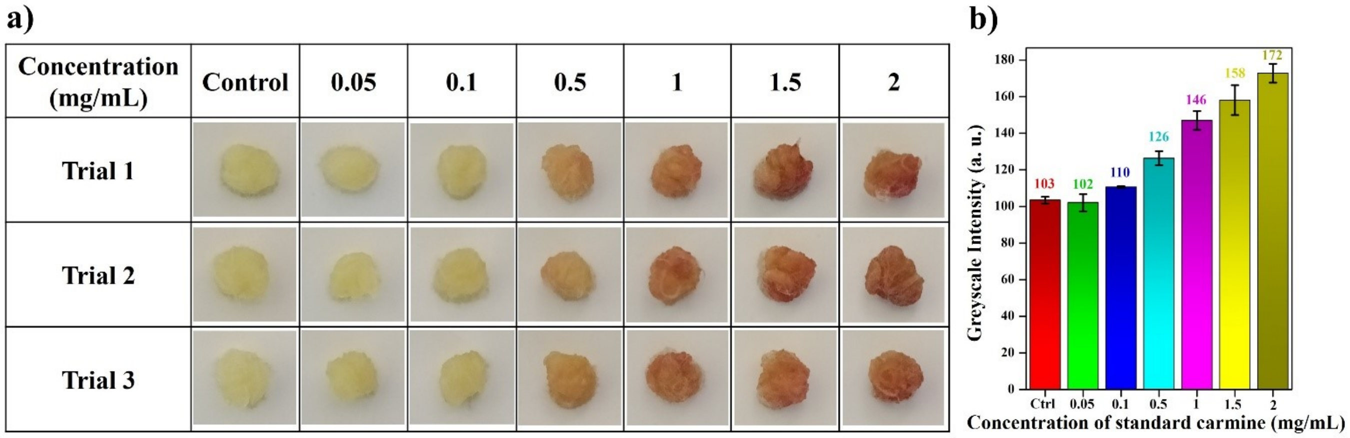

Supplementary Fig. 6: (a) Visual representation of cotton blobs (made of Flat cotton material) subjected to different concentrations of carmine. (b) Semiquantitative colorimetric analysis of cotton blobs using FIJI software, indicating a positive correlation between absorbance and concentrations, with error bars representing standard deviations.

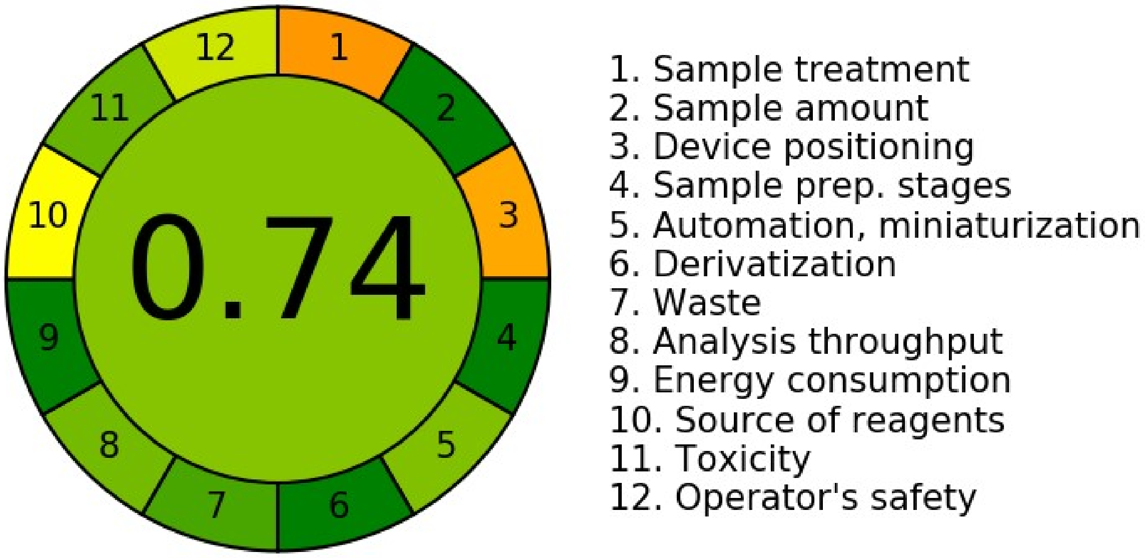

Supplementary Fig. 7: Green metric score of the proposed methodology of sensing carmine in lipstick using LoT device

Supplementary Table 6: Recovery analysis of carmine from spiked lipstick sample

**Recovery of carmine from spiked lipstick sample based on bulk testing (absorbance)**			
**Sample**	**Mean absorbance**	**Standard deviation**	**% RSD**
Spiked lipstick with 2 mg/mL carmine	0.952	0.064	6.71
Standard carmine (2mg/mL)	1.180	0.115	9.77
Recovery (%) = (Intensity of spiked sample/Intensity of standard carmine) *100<br>= (0.952/1.180) *100 = 80.7%			
**Recovery of carmine from spiked lipstick sample based on analysis using LoT device**			
**Sample**	**Mean intensity**	**Standard deviation**	**% RSD**
Spiked lipstick with 2 mg/mL carmine	174.188	3.93	2.26
Standard carmine (2mg/mL)	194.899	9.79	5.02
Recovery (%) = (Intensity of spiked sample/Intensity of standard carmine) *100<br>= (174.188/194.899) * 100 = 89.4%			

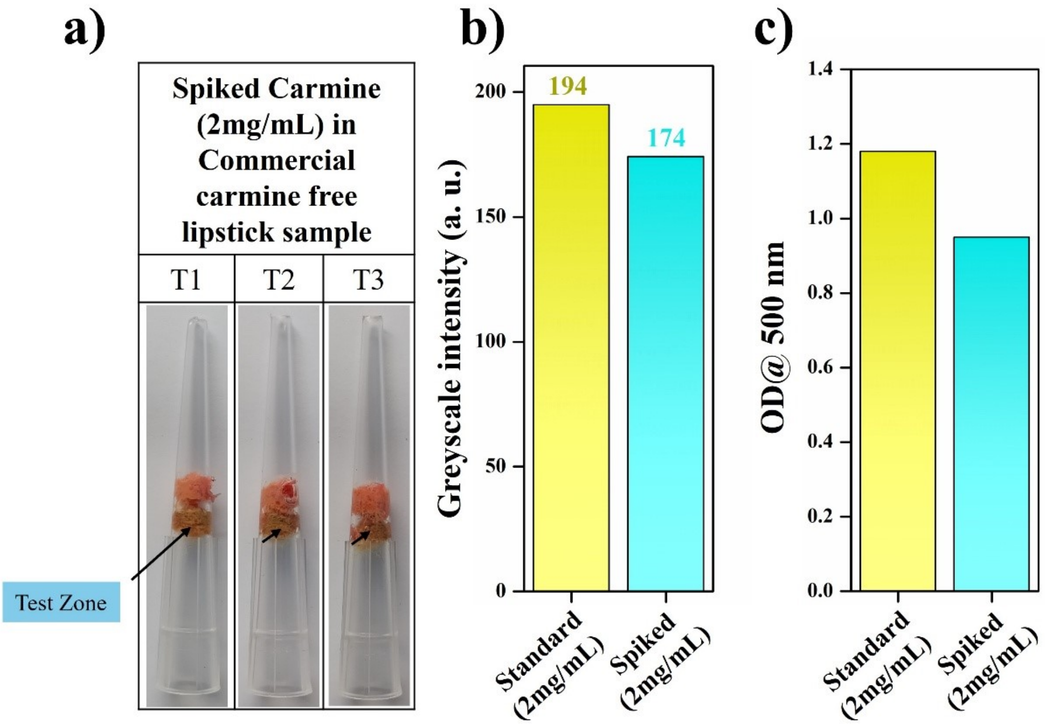

Supplementary Fig. 8: Recovery analysis of carmine from spiked lipstick sample a) Analysis of sample using LoT device b) Comparison between colour intensity of spiked sample and standard carmine sample (2 mg/mL) obtained using LoT device c) Comparison between absorbance of spiked sample and standard carmine sample (2 mg/mL) using UV-visible spectrophotometry
